# Ecosystem Health and Environmental Geography in the Belt and Road Regions

**DOI:** 10.3390/ijerph19105843

**Published:** 2022-05-11

**Authors:** Chunbo Huang, Yi Qin, Xixi Du, Jiawen He, Xin Fan

**Affiliations:** 1 State Key Laboratory of Biogeology and Environmental Geology, School of Geography and Information Engineering, China University of Geosciences, Wuhan 430074, China; huangchunbo@cug.edu.cn; 2 Center for Turkmenistan Studies, China University of Geosciences, Wuhan 430074, China; duxixi1110@cug.edu.cn (X.D.); hjw_1998@cug.edu.cn (J.H.); fanfx8@cug.edu.cn (X.F.); 3 School of Foreign Languages, China University of Geosciences, Wuhan 430074, China; 4 School of Public Administration, China University of Geosciences, Wuhan 430074, China

**Keywords:** the Belt and Road Initiative (BRI), environmental geography, ecosystem services, regional development, review

## Abstract

The “Belt and Road” Initiative (BRI), i.e., the official Chinese term for the “Silk Road Economic Belt” and the “21st Century Maritime Silk Road”, was proposed to share China’s development opportunities with BRI-related countries and achieve common prosperity. Though the BRI itself conveys rich social and economic connotations, ecosystem health and the environmental problems in the Belt and Road regions are scientific issues. In this study, papers relating to the ecological issues of the BRI between January 2013 and December 2021 were collected and analyzed via CiteSpace. We found that some ecological issues were involved with the environmental challenges posed by the BRI, whereas others were, to a certain extent, subjective assumptions. Accordingly, we identified and classified the limitations and constraints of those environmental views about the BRI. By emphasizing that scientific data is key to explaining the ecological problems, we advanced four prospects for ecosystem health and environmental geography studies in the Belt and Road regions: (1) Spatial analysis and monitoring technology for the environment; (2) Clarification of the characteristics and mechanisms of the ecosystem and environments; (3) A focus on the interaction between the economy and the environment; (4) Specific and targeted strategies and solutions to different environmental problems.

## 1. Backgrounds of the Belt and Road Initiative

The Belt and Road Initiative (BRI), proposed by Chinese President Xi Jinping in 2013, is an international initiative with vital implications for the economy, society, culture, and the environment [1]. Consisting of the “Silk Road Economic Belt” and the “21st Century Maritime Silk Road”, the BRI was inherited and developed from the ancient Silk Road that played an essential role in connecting the West with the East on various socio-economic levels with its spirit of peace, friendship, inclusiveness, openness, mutual benefit, and mutual learning for many centuries [2]. As China’s most ambitious long-term regional infrastructure project, the BRI primarily intends to provide unparalleled international economic cooperation opportunities and strengthen the trade and connectivity between Asia, Africa, and Europe with terrestrial and maritime routes [3]. To date, this initiative has officially involved 147 countries and 32 international organizations [4], with an estimated investment of USD 8 trillion by 2049. The BRI-related countries and regions constitute nearly 70% of the world’s population and account for more than 50% of global output [5]. It is projected that the population of Belt and Road countries (including China) will reach approximately 5.4 billion by 2030 [6]. It is claimed that the BRI will serve as a positive move toward cooperation among the Belt and Road regions, stimulating economic momentum in its member countries as well as other countries along the routes.

Under the BRI framework, China does not interfere in the internal affairs of countries in the Belt and Road regions, nor does it seek to play a dominant role [7]. Instead, the BRI intends to increase understanding and trust among BRI-involved countries and aims to achieve policy coordination, infrastructure connectivity, unimpeded trade, financial integration, and person-to-person bonds in Asia, Europe, and Africa [7]. To achieve these aims, the BRI has initiated a great number of projects including the extension of many industries, energy and resources development, electricity generation plants, road and rail infrastructure, natural gas pipeline construction, telecommunications, power grid construction and operation, poverty reduction, etc. [3]. With great theoretical and practical value, the BRI undoubtedly spurs a sharp acceleration in economic growth across the BRI member nations through bilateral collaboration and globalization. [8]. The economic benefits of the BRI are driven primarily by increased trade in goods, services, and resources, facilitated by reduced transportation costs and other trade barriers [9].

To promote regional trade flow and economic development, the BRI develops two main strategies: the development of surface physical infrastructure, which refers to the “belt”, and the improvement of maritime transportation, which is known as the “maritime silk road” [10]. These two strategies assemble numerous developed and developing economies into an open and inclusive network of cooperation, providing unprecedented opportunities for international enterprises to explore new markets in countries along the routes. The BRI has yielded fruitful results since it was proposed. From 2013 to September 2021, the cumulative value of trade in goods between China and BRI-involved countries amounted to USD 10.4 trillion [11]. In 2021, non-financial direct investment by Chinese enterprises in 56 countries along the Belt and Road routes amounted to USD 20.3 billion [12]. Furthermore, China–Europe freight trains have formed a grand channel for international trade spanning Eurasia. By the end of October 2021, a total of 73 routes for China–Europe freight trains had been launched, linking China with 175 cities in 23 European countries, and more than 46,000 trips had been made by these trains [11].

However, given the unprecedented dimensions of this initiative, many scholars have voiced concerns about its actual and potential negative ecological and environmental impacts. Some have maintained that current official BRI investment remains heavily concentrated on fossil fuels, traditional forms of transportation infrastructure, and climate-unfriendly sectors [13]. Dirty industries and environmentally harmful power generations were shifted abroad [14]. Still, others argued that the changes in China’s policy priorities toward a greener economy could, in fact, create a framework enabling China to outsource its polluting industries elsewhere while at the same time shifting its domestic economy to a new phase defined by the adoption of green technologies [15], regarding the BRI as simply a means for China to “export” its polluting model of development, with China’s partners as the pollution “dumping ground” for its own ecological civilization to denigrate the BRI [16].

Partly in response to growing international criticism, several Chinese ministries collectively issued policies on the “Green Belt and Road” to respond to the international trend of seeking green, low-carbon, and circular development [17]. However, in reality, most countries and regions along the BRI routes are in sensitive zones of climate and geological change, with complex natural and fragile ecological environments. The main parts of the Belt and Road regions are arid, semi-arid, or semi-humid areas that are fragile and sensitive with weak self-recovery abilities. Consequently, it is understandable that economic and social development may sometimes be accompanied by some inevitable impacts on the ecology and environment. However, some scholars used the consequences brought by the BRI as political attack tools and published their opinions and studies with obvious political intent in scientific journals. As the authors suggested, the political factors in the BRI’s green development can be overlooked in the discussion of a green BRI [18]. Regional ecosystem health and environmental problems are scientific issues that need to be addressed and researched scientifically and not to be politicized. Scientific approaches to environment-related issues should be advocated rather than politicizing those issues to denigrate the BRI. Therefore, we try to analyze the relevant papers published from 2013 to 2021 and review the ecological and environmental problems raised by the scholars. Meanwhile, we propose some suggestions and guidelines for research issues on ecosystem health and environmental geography in the Belt and Road regions in the future.

## 2. Quantitative Analysis of Environmental Studies Relating to the Belt and Road Initiative

### 2.1. Publications and Citations

To identify quantitative studies relating to the ecological issues of the Belt and Road regions, we performed a systematic search of scientific literature from the ISI Web of Knowledge (www.isiwebofknowledge.com (accessed on 18 April 2022)), which provides access to peer-reviewed studies. We conducted a pre-test, searching these databases for literature published between January 2013 and December 2021, and using the following search term combinations: (“belt and road” OR “silk road” OR “one road, one belt”) AND (“environment*” OR “ecosystem*” OR “ecolog*”). Finally, we obtained 623 qualified published references with related information on publication countries, years, authors, institutions, abstracts, keywords, journals, and their cited references.

After checking the search results, we found that “environment”, presenting polysemy, in most cases meant abstract background such as business environment or government-governance environment, which deviated from the ecological environment theme. Therefore, we adjusted the search term to give a more specific meaning to “environment”. Eventually, we obtained 470 requested records by retrieving the search term combinations (TS = “belt and road” OR “silk road“ OR ”one road, one belt”) AND (TS = “environmental” OR “eco-*” OR “pollution” OR ”emission” OR “ecolog*” OR “climat*”) and eliminating irrelevant articles. Meanwhile, we repeated the same operation on CNKI and obtained 108 English articles, only to find that they were completely covered by the ISI Web data.

**Figure 1:**
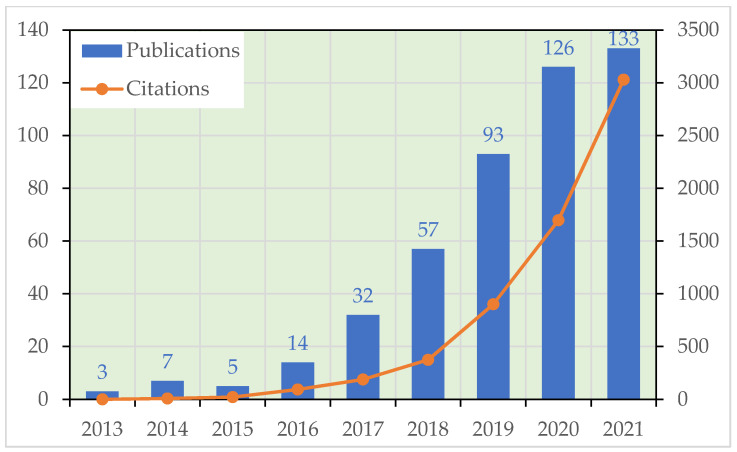
Dynamics of the annual number of published articles concerning ecological issues in the Belt and Road regions.

Since the BRI was first put forward in 2013, the ecology and environment of the “Belt and Road” regions have not obtained enough attention and there are few related publications from 2013 to 2015 (Figure 1). In 2016, the Chinese government released the 13th Five-Year Plan for ecological environment protection, which set the goals for a more environmentally friendly lifestyle, a considerable reduction in the discharge of pollutant emissions, effective control of environmental risks, and a sounder ecological system. As the concept of ecological civilization is deeply rooted in people’s hearts, promoting green Belt and Road to lead the international trend of green, low-carbon, and circular development is an inevitable and effective way to boost sustained and sound economic growth. In May 2017, relevant departments of the Chinese government jointly issued the “Guidance on Promoting a Green Belt and Road” [19] to further promote the green development of the Belt and Road regions. Therefore, the number of related references has grown exponentially since 2017 (Figure 1). In recent years, environmental discussions in the Belt and Road regions have been sprouting up. As the topic becomes more attractive, the number of citations also increases yearly (Figure 1), which confirms the value of the research.

### 2.2. Interested Countries and Regions

**Figure 2:**
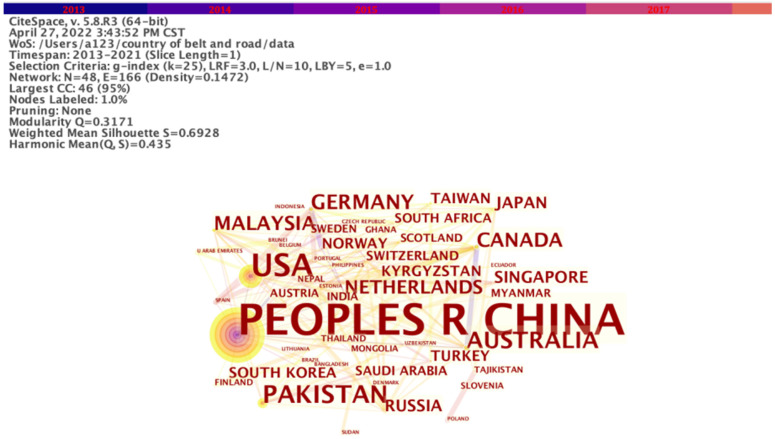
Co-country network of ecological issues in the Belt and Road regions.

CiteSpace is a scientometric software that can be used to generate knowledge domain visualization and detect emerging trends in scientific literature [20,21]. The retrieved articles were imported into CiteSpace 5.8.R3 for further analysis following the process: Time Slicing set as “From 2013 to 2021”, Link set as Cosine, and Selection Criteria set as g-index = 25. After processing, we obtained the merged co-country network map with 48 notes and 166 links (Figure 2). Besides China, the main countries concerned about the ecological and environmental issues of the Belt and Road regions can be divided into 2 categories.

**Figure 3:**
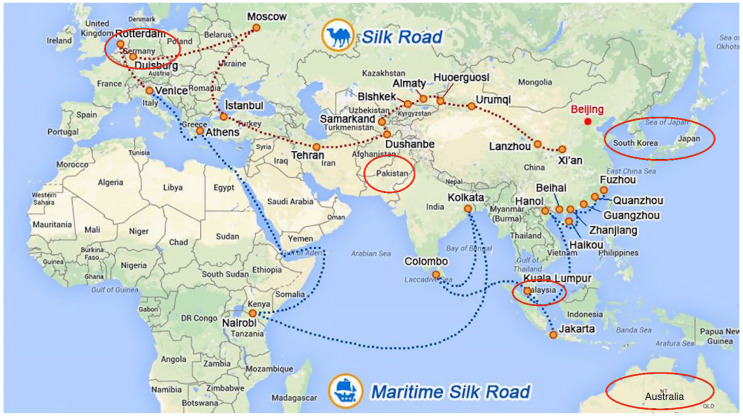
Road map for the “One Belt One Road” and location of interested regions (Red oval is used to show the location of interested region).

The first category consists of countries along the route, particularly those near the first stop of the BRI, mainly in South and Southeast Asia (Figure 3). These nations act as the hubs for “the oceans and continent” trade networks of the BRI such as Malaysia, Singapore, and Pakistan, etc. The ecological problems there are more complex, involving both marine and terrestrial ecosystems, which leaves room for further exploration. The European countries at the terminus of the route, such as Germany and the Netherlands, are characterized by an eco-friendly consciousness and are always alert to potential environmental dangers.

The other is the countries that geographically deviated from the six economic corridors of the Belt and Road Initiative such as the United States, South Korea, Japan, and Australia. Although these countries are not in important areas alongside the Belt and Road regions, they have close economic ties with China, always keeping a watchful eye on China’s dynamics and development. Consequently, the BRI policy has become a topic of interest for them.

### 2.3. The Evolution of Research Hotspots

**Figure 4:**
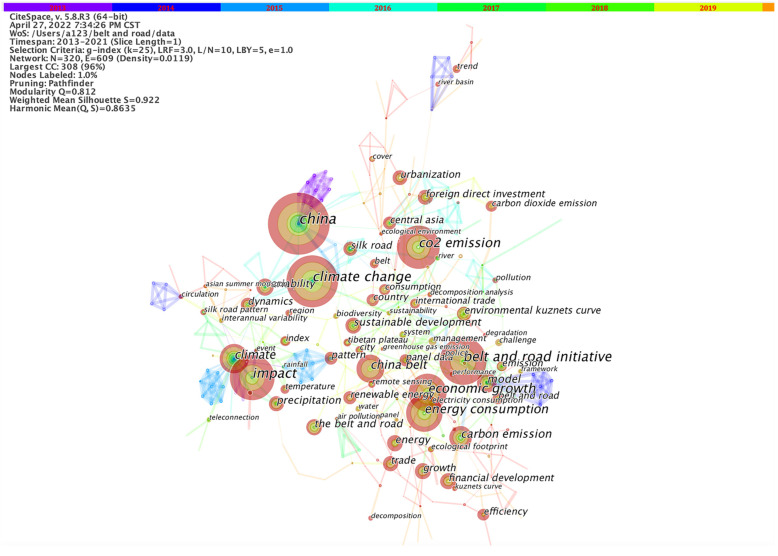
High-frequency keywords scatter view of ecological issues of the Belt and Road.

**Figure 5:**
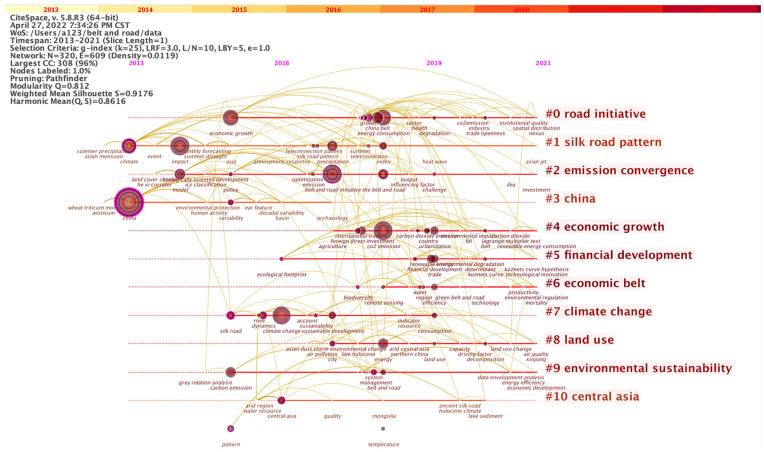
Keywords timeline view of ecological issues of the Belt and Road.

We used CiteSpace 5.8.R3 to analyze the network of co-occurring keywords by setting pathfinder pruning (Figure 4). The knowledge network shows 320 notes and 609 links, resulting in 22 clusters. The top 11 clusters contain more than 10 keywords, displaying diverse research theses, study objects, and research methods (Figure 5). The modularity Q value is 0.812 and the silhouette S value is 0.9176, which mean that the structure of our cluster data is clear and the results are reliable.

The clustering of keywords reflects the change in research hotspots and shows the evolution of ecological environment research along the Belt and Road [22] routes. Cluster #0 (road initiative) refers to the Belt and Road Initiative; its main content is to unscramble the target and empirically examine the influence of this policy on the world. Due to the continuous advancement of this initiative, policy research and empirical study will carry on. Cluster #1 (silk road pattern) is a meteorological teleconnection pattern covering most domains along the ancient Silk Road and exerting a significant impact on climatic anomalies over a broad area of the Eurasian continent. Cluster #2 (emission coverage) focuses on greenhouse and other gas emissions in construction and freight transportation. In the context of low-carbon development, carbon emission transfer and carbon emission efficiency have become the hot spot of research. Cluster #3 (China) implies China, as the initiator of this policy, was the core object of study before 2017. Cluster #4 conveys the influence of economic activities such as commodity trade and foreign investment on the ecological environment. Cluster #5 describes a more specific relationship between financial flow and the environment. Cluster #6 (economic belt) is an abbreviation of the belt economic zone, emphasizing the unique geographical advantages of the regions along the Silk Road such as demographic structure, productivity level, resource endowment, etc. Cluster #7 indicates how climate changes affect regional development. Unlike Cluster #1 which uses remote sensing technology to predict and monitor climate change, Cluster #7 focuses on the effects of climate change and has stagnated in 2019. Cluster #8 refers to land use. Although this topic appeared relatively recently, its extensive scope highlights its research significance. The changes in the landscape and soil environment prompted by land use are due to the primary interference by human activity in the natural environment. Cluster #9 stresses that environmental sustainability has become a curtailed evaluation index for the projection of the BRI. Cluster #10 reveals that Central Asia was once the center of attention for its glorious history of the ancient Silk Road Period and its fragile ecological environment. In terms of the vitality of keywords, the ecological research of the Belt and Road Initiative presents the following characteristics: using high-tech solutions, intertwining with the economy, excavating policy connotations in-depth, and analyzing human activities.

### 2.4. The Development Venation of Research

**Figure 6:**
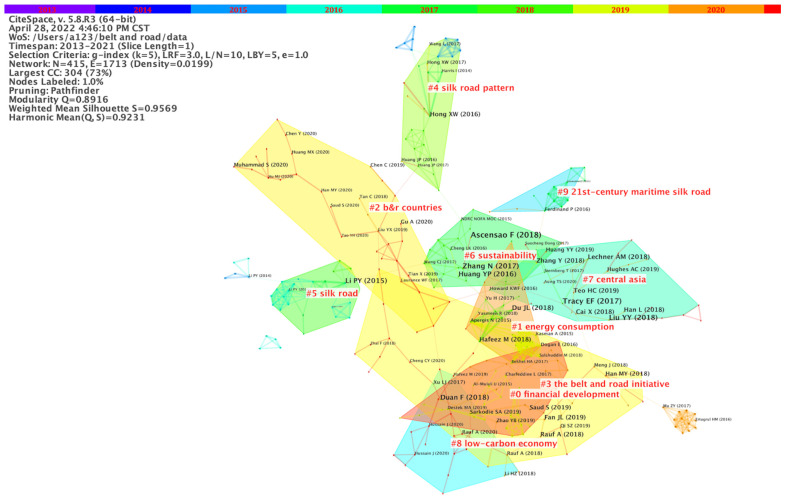
Literature co-citation cluster network of ecological issues in the Belt and Road regions.

The visualized analysis of the references can reveal the intellectual base and research front of the research. The intellectual base is the co-citation venation, and the research front is the derivative of the knowledge base and represents the emerging research direction or subject [23]. We still set the Selection Criteria as g-index = 25 and pruned the network using the pathfinder. The results are shown in Figure 6.

After calculation, 62 clusters were finally obtained. We selected the 10 largest clusters with tight relationships. Figure 6 shows that the BRI (Cluster #3) serves as an intermediary, linking different topics. Among them, financial development (Cluster #0) and low-carbon economy (Cluster #8) are closely tied up with the Belt and Road Initiative, reflecting the core pursuit of the Belt and Road construction. Energy trade is also an important issue in the Silk Road region. During the process of energy cooperation, the importance of Central Asian countries cannot be ignored and environmental sustainability must be taken into account.

**Figure 7:**
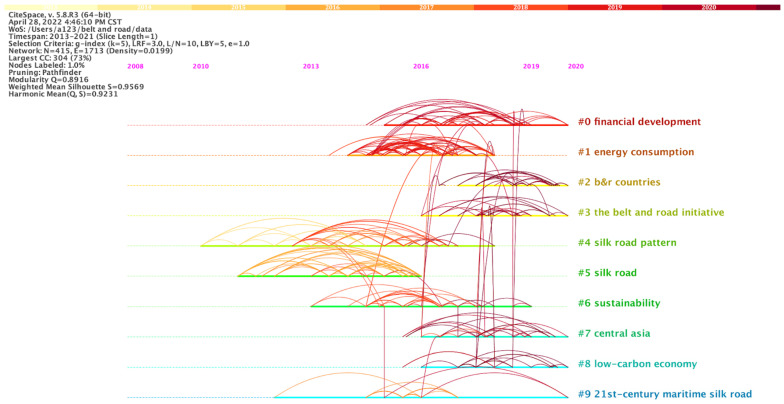
Literature co-citation timeline view of ecological issues in the Belt and Road regions.

Figure 7 shows the evolution of the research topics. The Belt and Road ecological environment study began with the three clusters—“silk road pattern”, “silk road”, and “21st Century Maritime Silk Road”. The original subjects of the study are monsoon activities, soil and water management, and the marine environment, respectively [24,25,26,27]. Natural ecological changes and the impact of human activities on the environment are the starting point of the initial research. Between 2013 and 2016, the number of clusters #0, #1, and #6 increased dramatically. During this period, research on land creation shows the strongest citation bursts, which means that the empirical study on the interaction between economic development and the ecological environment has become the focus of discussion [28]. At the same time, people began to reflect on the negative impacts of economic benefits on the ecological environment and realized the importance of environmental sustainability. The citation burst revealed the research hot spot for a period of time, suggesting that the research changed direction [20]. The latest three citation bursts [29,30,31] documented energy investment, regional temperature changes, and the environmental Kuznets curve, suggesting a future trend of ecological environment research along the Belt and Road routes: (1) climate change caused by atmospheric activity and monitored by remote sensing; (2) establishing an environmental assessment mechanism; (3) evaluating economic activity from the perspective of the environmental costs, especially energy consumption; (4) exploring the relationship between economic development and the ecological environment.

## 3. Ecological Problems in the Belt and Road Regions

Many environmental challenges of the BRI have been proposed by scholars. We summarized some of the main challenges according to environmental elements and subdivided them into biotic, water, soil, atmospheric, and geological environments. The views of those scholars on the environmental challenges will be listed according to this classification.

### 3.1. Biotic Environment Challenges

#### 3.1.1. Biodiversity Loss

Infrastructure projects implemented by the BRI might necessarily pass through ecologically fragile regions and key biodiversity areas [32]. Thus, the planned and probable development of these infrastructure projects along the routes may pose a significant risk to biodiversity.

##### Terrestrial Biodiversity Loss

The BRI’s infrastructure project and its impacts are the key drivers of biodiversity losses [33]. The BRI will cross several terrestrial biodiversity hotspots, wilderness areas, and other key conservation areas [15]. Therefore, the impacts of infrastructure expansion are likely to create obvious threats to biodiversity [32,34,35]. Roads, for example, may have negative impacts on ecological health and the environment, such as habitat loss, fragmentation, invasive species, and illegal activities such as poaching, leading to biodiversity losses [33]. Increased wildlife mortality, restrictions on animal movement, pollution (chemicals, noise, light), and the spread of invasive species caused by road construction under the BRI could have negative impacts on terrestrial biodiversity [36]. Raw material extraction for building roads or supplying resources for new population centers (e.g., power supply) [37] and increased access to natural resources due to greater accessibility along the route [38] could also affect biodiversity.

##### Marine Biodiversity Loss

Including sea routes, the BRI will also cross several marine biodiversity hotspots. In the marine environment, increased marine traffic exacerbates the movement of invasive species and pollution, which could have disastrous consequences for marine biodiversity [33]. Port infrastructure and an increase in shipping lane traffic associated with the maritime component of China’s BRI will lead to overfishing and noise pollution, which are likely to threaten marine species and reduce marine biodiversity [39]. According to the research, over 400 threatened marine species, including mammals, could be affected by port infrastructure, and over 200 threatened species are at risk from an increase in shipping traffic and noise pollution [40].

#### 3.1.2. Habitat Fragmentation

With the BRI vigorously implementing infrastructure projects, linear infrastructure in particular, such as roads and railways, is considered one of the leading proximate causes of habitat fragmentation in terrestrial ecosystems [39]. In order to create a transportation corridor, roads or railways could be built at the expense of habitat destruction, which causes habitat loss and fragmentation, leading to dramatic landscape transformation and loss of the ability to support healthy ecosystems and populations of plants and animals [36,41]. Port infrastructure development is likely to affect key coastal marine habitats (coral reefs, mangroves, seagrasses, and saltmarshes) and threatened marine species [40].

#### 3.1.3. Wildlife Populations Decrease

Infrastructure development, in particular, linear infrastructure such as roads and railways that could bring about hunting and traffic disturbances, drastically caused the decline and extinction of wildlife populations [39]. The construction of transportation infrastructure under the Belt and Road Initiative could contribute to the highest rates of wildlife–vehicle collision and poaching. The situation is exacerbated for wildlife that are attracted to open flyways and paths, further increasing the risk of wildlife–vehicle collisions [42]. Roads and minor roads that penetrate frontier landscapes, in particular, can increase both legal and illegal hunting of wildlife. Improved transportation networks can facilitate the movement of illegal wildlife trafficking [32,41]. In addition, the barriers created by roads and railways might endanger wildlife populations by disrupting migratory routes, splitting populations, and thus reducing genetic variability [43]. Noise pollution from trains (freight and passenger) has also been recognized as harmful to the health of wildlife populations [41].

#### 3.1.4. Deforestation

The development of infrastructure will likely have negative consequences for forest resources. Nearly all BRI infrastructure projects will have associated direct impacts on the environment such as the clearance of vegetation [44]. In detail, road and railway construction under the BRI opens up frontiers for settlement while increasing market access for farmers and ranchers to forests, thus increasing deforestation [32,41]. In tropical forests, new openings for roads and other linear infrastructure will likely increase illegal logging and fires by facilitating access to hitherto remote regions, thus causing forest loss and having an impact on forest ecosystems [36].

Moreover, the BRI’s high expenditure on infrastructure construction by accelerating trade and transportation networks may also have increased the risk of biological invasion [45].

### 3.2. Water Environment Challenge

#### 3.2.1. Challenge on Surface Water

##### Water Pollution

With the implementation of BRI infrastructure projects, increasing accessibility will further result in novel developments associated with urbanization, extractive industries, and agriculture, which could generate water pollutants [44]. The number of new or upgraded roads or rails will increase, which could cause corrosion, exhaust emission, trash, and other pollutants to be washed into waterways, thus creating water pollution, especially during heavy rainstorms or floods when the drainage capabilities of ditches and soil are exceeded [41]. Mineral exploitation may also cause water pollution [32]. With advances in agricultural cooperation, pastoralism has developed rapidly, with intensive livestock systems as one of the manifestations. Intensive livestock systems are also likely to produce a large number of polluted watercourses [46].

##### Water Shortage

Some Belt and Road regions, such as Central Asia, have long suffered from water shortages. The new “Silk Road Economic Belt” may well bring jobs and prosperity to Central Asia, thereby alleviating some of the social concerns. However, it will also accelerate population growth and demand for water, further exacerbating the water shortage problem [47]. The agricultural development brought by the BRI might increase the irrigation demands, thus increasing water consumption and leading to water shortage [1]. The growth of manufacturing, food processing, and nuclear fuel processing might also accelerate water consumption [48]. Additionally, the infrastructure will affect almost all of Eurasia’s largest river systems, such as the Mekong, and may exacerbate water stress in regions such as Central Asia [9].

#### 3.2.2. Challenge on Groundwater

With the implementation of the BRI infrastructure projects, groundwater pumping rates will likely rise due to demand for infrastructure (e.g., concrete), mining, and agriculture. Severe groundwater depletion could also lead to a shift in water flows from the surface to the underground, resulting in contamination by metals, nutrients, and pesticides in groundwater reserves, which may limit future groundwater use and endanger little-known subterranean biodiversity [1].

### 3.3. Soil Environment Challenges

#### 3.3.1. Soil Erosion

The soil has deteriorated due to severe water contamination and scarcity caused by BRI infrastructure projects [49]. The construction and maintenance of BRI transportation and energy infrastructure projects, such as roads and hydropower, may cause soil erosion [9]. Meanwhile, agricultural cooperation projects under the BRI might also increase erosion and soil loss [32] such as the intensive livestock systems in Central Asia [46].

#### 3.3.2. Soil Pollution

The BRI’s infrastructure projects in the areas of transportation and energy, such as railways, airports, pipelines, and power lines, could also cause soil pollution [36] and transform the stable and fertile soils [44].

### 3.4. Atmospheric Environment Challenges

Some scholars proposed that the BRI could increase global greenhouse gas (GHG) emissions because of the heaving tendency of energy consumption, gross fixed capital formation, economic growth, economic freedom, financial development, logistics operation, transportation, industrialization, urbanization, and the clearing of forests for roads and railway lines brought about by the BRI [50,51,52,53,54,55,56]. BRI-associated countries have spurred energy utilization due to its vital role in the economy to increase production, which, in turn, caused a surge in carbon dioxide emissions [51,57]. Construction and maintenance of transportation infrastructure under the BRI and further Chinese investment in coal-fired power plants could also increase GHG emissions [58]. Cement production, which is mainly used to build roads, also emits large amounts of GHG. The increasing number of power plants could also contribute to greenhouse emissions, such as nitrogen oxides and sulfur dioxides [54]. Furthermore, large investments in pipeline infrastructure will increase the rate at which oil and gas reserves are exploited, further increasing greenhouse gas emissions worldwide. The increased shipping associated with the BRI might also contribute to this impact [36]. BRI energy projects with an excessive adoption of fossil fuel and renewable energy could also contribute to the soaring emissions of greenhouse gases and other pollutants [41,49]. Extending the supply chain by streamlining exports to Belt and Road countries will lead to the growth of China’s energy-intensive industries (such as mining, iron, and steel) that could, in turn, accelerate energy combustion and increase greenhouse gas emissions [58]. Meanwhile, in terms of agricultural cooperation, intensive livestock systems could produce high levels of carbon dioxide and methane [46]. In addition, microbial decay and the burning of plants and organic matter may also emit carbon dioxide into the atmosphere [59].

### 3.5. Geological Environment Challenges

#### 3.5.1. Overexploitation of Resources

The large amounts of raw material needed to support the expansion of BRI infrastructure will boost the extraction and utilization of natural resources, such as sand and limestone for the production of concrete and cement, and fossil fuels for BRI energy projects [32,36,55].

#### 3.5.2. Impact on the Coastline

The construction of industrial, agricultural, and aquaculture parks as well as new ports is impacting the coastline via sedimentation, the destruction of biota, and pollution [60]. Additionally, coastal ecosystems, as the bridge linking the maritime and terrestrial worlds, could be threatened if subjected to increased shipping, new port developments, and reclamation as well as pollution [1].

In conclusion, based on the above content, we summarize the biotic, water, soil, atmospheric, and geological environment challenges proposed by scholars, as well as the impacts and the corresponding causes (Table 1).

## 4. Limitations and Uncertainties of these Environmental Views

### 4.1. Perspectives of Environmental Ethics

#### 4.1.1. Anthropocentrism and Nonanthropocentrism

Environmental ethics aims to explain the relationship between humans and the environment and examines both anthropocentrism and nonanthropocentrism (including biocentrism and ecocentrism) [61]. The term “anthropocentrism” was first coined in the 1860s amid the controversy over Darwin’s theory of evolution, to represent the idea that humans are the center of the universe [62]. Anthropocentrism considers humans as the most significant life form in the universe and other forms of life to be important only to the extent that they can be useful to humans. Anthropocentrism calls for the restrained use of nature for the consumers’ own interests in order to take the best possible (long-term) advantage of nature’s resources and ecosystem services [63]. Nonanthropocentrism is just the denial of anthropocentrism. Nonanthropocentrism argues that the nonhuman world has value for its own sake, rather than only existing to directly or indirectly serve human interests [64]. It claims that humans should respect nature rather than the mere use of nature, thus human interests and needs cannot be met at the expense of the nonhuman world [65].

#### 4.1.2. Anthropocentrism versus Nonanthropocentrism: Which Is the Mainstream?

Some scholars criticized the development of the BRI from the perspective of nonanthropocentrism, claiming that the existence of the nonhuman world does not directly or indirectly serve the interests of human beings and that the advancement of the BRI should be suspended to ensure that the environment is not damaged. However, development is needed for better conservation, and wise utilization is better than blind protection. It is relatively unwise to sacrifice human and material well-being for the greater health of the nonhuman world. Human beings should develop first and then development can bring new high-tech technology to protect the non-human world [6]. On the premise of fully protecting the nonhuman world, allowing the environment to be of value to humans is the key to realizing the sustainable development of humans and the environment.

Meanwhile, other researchers argued that anthropocentrism is inevitable and even benign for the aim of environmental conservation [66,67]. Some environmental economists maintain that ecosystems, natural resources, and species communities are valuable only because they are valuable for consumers. However, this anthropocentric view is not equivalent to opposing nature protection. It rather implies that nature protection ought to be derived from and limited to the value of nature to humans. As many BRI-related nations are rich in energy reserves, natural resources cooperation widely occurs in the Belt and Road regions. The exploitation and export of natural resources (such as coal, oil, natural gas, and other fossil fuels) can bring economic benefits to these resource-rich nations. In the process of exploitation people have recognized that natural resources guarantee human existence and excessive exploitation would cause irreversible damage to nature. Therefore, with the promotion of the green BRI, people have begun to develop renewable resources (such as nuclear energy, solar energy, wind energy, etc.) and rationally utilize natural resources in order to protect nature and achieve a unity of environmental and socio-economic benefits. This is a manifestation of anthropocentrism that can and should serve as a powerful motivator for the protection of nature since the best reason to protect ecosystems is that these ecosystems constitute the ‘life-support system’ for humans so it is in humans’ best interests to protect nature [67]. Taking resources from the nonhuman world and calling for unwasteful use of those resources are in line with the ambitions of anthropocentrism, which guarantees natural protection in order to benefit humanity as a whole [67].

It is anthropocentrism that has become the norm for resolving environmental issues and enhancing environmental protection. With the help of anthropocentrism and a clear distinction between legal and illegal human interests, the development of the BRI and its environmental challenges could be properly studied. However, in the process of developing under the guidance of anthropocentrism, it is crucial to realize that the relationship between man and nature is an organic unity of inter-communication, interaction, mutual benefit, and harmonious coexistence. Human beings have a duty and obligation to respect the right to exist of other species in nature. On the basis of maintaining an ecological balance, human beings should develop nature in a rational way, regulate their behavior towards nature, limit modes of production and consumption within a range that the ecosystem can bear, and advocate for the utilization of nature while still loving, respecting, and protecting it.

### 4.2. Ignoring the Inevitable Factors

As some BRI countries are sensitive to invasive species and have limited financial resources and coping capabilities, they will be more vulnerable to biological invasions, which is, in fact, a long-term and inevitable natural phenomenon. Biological invasions not only affect the ecosystem structure and function, but they also have economic and environmental impacts [68]. The process of biological invasion can be described as the introduction of species in biogeographic areas outside their native range, with harmful consequences to the invaded ecosystem [69]. It could be done in two ways: natural and artificial. Natural invasion is caused by the natural migration of plant seeds or animal larvae, eggs, or microorganisms through the flow of wind or water, or by insects or birds, whereas anthropogenic biological invasion occurs through the development of the economy and the upgrading of means of transportation. For example, some diseases, insect pests, and harmful pathogens mostly infiltrate through international trade and transportation activities. Once invasive species are established in relatively stable ecosystems, they will cause ecological problems known as “biological pollution”, affect human activities, endanger the biodiversity of local communities, and cause enormous economic losses [70]. Although anthropogenic biological invasion is caused by human action, humans do not have malicious intent to introduce invasive species. The implementation of the BRI infrastructure projects, which has led to a more comprehensive transportation network and more frequent trade among the BRI countries, may unavoidably trigger biological invasions as well. The inevitable factors of biological invasions should not be ignored.

### 4.3. Unreasonable Substitution without Sufficient Support

In some studies, scholars only documented the impacts of other infrastructure construction on the environment and substituted them unreasonably without specific investigation and evaluation, believing that the implementation of the BRI project would also cause those impacts on the environment. For instance, Hughes [32] pointed out that the development of the BRI generates some potential risks for biodiversity. Some scholars proposed that the BRI would bring increased roadkill [71], increased wildlife trafficking resulting from increased regional connectivity [72], and habitat fragmentation due to road construction [73]. Although these potential environmental risks were proposed, none of the literature cited by the author was BRI-related projects. Meanwhile, actual cases and accurate data were not provided to analyze the Belt and Road regions. Consequently, without specific investigation and evaluation, it is not rigorous to directly apply this kind of environmental risk to the BRI. In fact, proper planning of the roads and railways in the Belt and Road regions could avoid biological reserves and habitats. Carefully designed protection measures of construction projects can prevent wild animals from entering the roads at will, thus avoiding roadkill. Additionally, with the quality of people greatly improved, the rate of wildlife trafficking could also be reduced. Consequently, a concrete analysis of concrete problems must be advocated rather than directly bringing previous research results into current environmental research on the BRI.

There are also some scholars who only mentioned the environmental problems the BRI infrastructure projects would cause without using scientific data to support their arguments, let alone explore the mechanisms behind these possible environmental problems. For instance, the authors in [41] pointed out that “Deforestation caused by land-use changes following changes in transport costs can dramatically exacerbate environmental risks”. They proposed that deforestation can exacerbate environmental risks without elaborating why deforestation can aggravate the environment and what kind of risks it will bring. The authors in [9] stated that “BRI infrastructure will affect almost all of Eurasia’s largest river systems, such as the Mekong, and may exacerbate water stress in regions like Central Asia”. There is, again, no data to support their predictions, with a reduction in water volume in those river systems after the implementation of BRI infrastructure projects unreported.

The approach in [74] could be advocated, instead. The author cited specific data to show that some energy-intensive projects along the BRI would emit carbon dioxide and other greenhouse gases, thus affecting the atmospheric environment. In [74], the author described that “In BRI projects, 65 percent of the total energy generation funds are invested in coal-based power plants. China is building 240 coal-based power plants in 25 BRI countries, which contains an installed capacity of 251 Gigawatts. Furthermore, the proportion of energy-intensive CO2 emissions in BRI economies is about 80%, indicating the crucial contributions of the energy sector to environmental degradation”.

## 5. Prospects for Ecosystem Health and Environmental Geography in the Belt and Road Regions

In view of the potential environmental challenges proposed by scholars and the deficiencies in existing research described above, the focuses for future study are recommended. They could be, but not limited to, the following research directions.

### 5.1. Spatial Analysis and Monitoring Technology for the Environment

A lack of access to comprehensive and up-to-date data is a key limitation of conducting in-depth studies on the ecology and environment of the BRI. Spatial analysis and monitoring technology can provide large-scale monitoring tools to obtain data on the status and changing trends of ecological and environmental quality. These scientific data can be used to judge the level of environmental pollution and quality, objectively evaluate the current main ecological and environmental problems, and serve as environmental management. Consequently, to better and more objectively assess the ecologies and environments of the Belt and Road regions, more research needs to be conducted using spatial analysis and monitoring technologies.

### 5.2. Clarification of Characteristics and Mechanisms of Ecosystems and Environments

#### 5.2.1. Ecosystem Health

Ecosystem health, a kind of functional manifestation of ecosystem operation, can ensure the ecosystem develops well [75]. First put forward by Rapport in 1998, ecosystem health means that an ecosystem possesses stability and sustainability, that is, it possesses an ability to keep its organization, regulate itself, and restore coercion [76]. Ecosystem health is a comprehensive characteristic of the ecosystem, which can reflect the regional ecological environment from multiple perspectives. It can be described as a comprehensive, multiscale guiding framework in the evaluation of ecosystem vigor, organization, and resilience [77,78]. This evaluation mechanism of the ecosystem, comprising vigor (activity, metabolism, or primary productivity), organization (the diversity and number of interactions between system components), and resilience (the ability to maintain its structure and pattern of behavior in the presence of stress) [77,79], determines the assessment framework of ecosystem health and affects the identification of the regional environment. A systematic study of regional ecosystem health, therefore, can not only clarify the characteristics and spatial-temporal changes in regional ecological environments in the Belt and Road regions from multiple perspectives, but also contribute to the understanding of the driving mechanisms of regional ecological environmental change, providing a scientific basis for environmental protection under the BRI framework.

#### 5.2.2. Environmental Impacts of the BRI

The atmospheric, water, soil, geological, and biotic environmental impacts of the BRI proposed by scholars have been summarized and discussed. It is necessary to understand the environmental impacts of the BRI as a prerequisite for effective strategies, which encourages the sustainability of environmental, social, and economic development. Consequently, the driving factors of these effects (such as the factors affecting the carbon emissions by the transportation industry in countries in the BRI) and the direction and extent of these effects are both worthy of further discussion.

#### 5.2.3. Energy Transformation for the BRI

With the in-depth development of the BRI, the demand for energy in its construction becomes increasingly urgent, and thus energy transformation and energy cooperation, which are highly relevant to climate change, will be another focus. In the BRI cooperation, countries along the routes should pay special attention to working with other countries to achieve carbon and emission reduction targets. How to increase the proportion of new energy and gradually reduce the proportion of traditional energy by means of technological progress is a major problem that China and all BRI partners need to discuss and resolve in the process of energy transformation and cooperation.

### 5.3. Focus on the Interactions between the Economy and the Environment

#### 5.3.1. Ecosystem Health and Regional Development

A healthy ecological environment, such as low air pollution and rich biodiversity, can provide important production resources for regional development. As long as an ecosystem is healthy and well-protected, ecological and green industries can be developed to realize the economic value, thereby boosting regional development. Ecosystem health is a prerequisite for regional development. Whether the ecological environments of the Belt and Road regions could support regional development and how to support it remain a largely unexplored area. Investigating the relationship between ecosystem health and regional development among these regions is the foundation for guaranteeing ecosystem health in order to promote regional development as a whole.

#### 5.3.2. Development and Protection

It is essential for future studies to attach importance to the relationship between development and protection. The relationship between economic development and the ecological environment should not be regarded as a zero-sum game. Does economic growth inevitably lead to ecological destruction? Is there a balanced model to achieve a “win-win” scenario for economic growth and environmental protection? Can this model be applied to future developments in the Belt and Road regions? These issues must be probed and clarified for further research.

### 5.4. Specific and Targeted Strategies and Solutions to Different Environmental Problems

Along with advances in BRI construction, infrastructure construction, export supply chains, and energy-intensive industries would surge, which could result in an increase in some unavoidable environmental problems such as greenhouse gas emissions, water pollution, and so on. Therefore, it is advisable to propose constructive strategies and solutions to environmental problems from different perspectives. Due to the different levels of development, financial status, sensitivity, and responsiveness to environmental changes in the Belt and Road regions, addressing environmental problems involved in the BRI is complex and multi-scaled. At the same time, the BRI infrastructure types could be divided into communication, transport, energy, and economic infrastructure and activities, all of which have different impacts on the environment. Hence, it is significant to propose specific strategies and solutions to the environmental problems caused by various types of infrastructure in different regions. This could be carried out by international organizations or non-government organizations or by countries, enterprises, or individuals.

## 6. Conclusions

Due to the harsh ecological environment of the Belt and Road regions, it is understandable that the quest for economic and social development under the BRI brings with it some inevitable impacts on the ecological environment. Some scholars have voiced concerns about the actual and potential negative ecological and environmental impacts of the BRI. In this review, the visualization tool, CiteSpace, was applied to bibliometric analyses and then the views of scholars on the environmental challenges were summarized and listed according to the five categories of environmental elements. Nevertheless, we found that some environmental challenges were related to the BRI projects, whereas others were somewhat subjective. Correspondingly, we discussed the limitations and constraints of those environmental views.

By emphasizing the scientific approach and objectivity as key to explaining ecological problems, we proposed four suggestions for future research on ecosystem health and environmental geography in the Belt and Road regions: (1) Spatial analysis and monitoring technology for the environment that could provide scientific data for researchers to objectively evaluate the current main ecological and environmental problems. (2) Clarification of the characteristics and mechanisms of ecosystems and environments. A systematic study of ecosystem health can not only clarify the characteristics and spatial-temporal changes of the ecological environment from multiple perspectives but also contribute to an understanding of the driving mechanisms of ecological environmental changes in the Belt and Road regions, thus providing a scientific basis for environmental protection under the BRI framework. (3) Focusing on the interactions between the economy and the environment, which is the foundation for guaranteeing ecosystem health in order to promote regional development as a whole. (4) Specific and targeted strategies and solutions, which are of great significance to slove complex and multiscale environmental problems in the regions. This review can provide a reference for ecosystem health and environmental geography studies in the Belt and Road regions and contribute to securing life-supporting ecosystem services for human benefit.

## Figures and Tables

**Table 1:** The category, impacts, and causes of environment challenges in the Belt and Road regions.

Challenges Category	Impacts	Causes			Representative References
		Transportation	Energy	Agriculture	
Biotic Environment Challenges	Terrestrial Biodiversity Loss	Poaching; increased wildlife mortality; the spread of invasive species; restrictions of animal movement; pollution (chemicals, noise, light)	Pollution (chemicals, noise, light); raw material extraction		Tracy et al., 2017 [15]; Hughes, 2019 [32]; Lechner et al., 2018 [33]; Aung et al., 2020 [34]; Huang and Li, 2020 [35]
	Marine Biodiversity Loss	Construction of ports; increased marine traffic; invasive species; noise pollution	Pollution (chemicals, noise, light)	Overfishing	Lechner et al., 2018 [33]; Ng et al., 2020 [39]; Thurschwell et al., 2020 [40]
	Habitat Fragmentation	Roads and railways construction			Ng et al., 2020 [39]; Ascensão et al., 2018 [36]
	Wildlife Populations Decrease	Hunting; poaching; wildlife-vehicle collision; wildlife trafficking; noise pollution			Ng et al., 2020 [39]; Losos et al., 2019 [41]
	Deforestation	Illegal logging and fires			Hughes, 2019 [32]; Losos et al., 2019 [41]
Water Environment Challenges	Surface Water Pollution	Exhaust emission, trash and other pollutants caused by new or upgraded roads or rails	Metal corrosion; exhaust emission; mineral exploitation	Intensive livestock systems	Hughes, 2019 [32]; Losos et al., 2019 [41]; Foggin et al., 2021 [44]
	Surface Water Shortage		Nuclear fuel processing	Irrigation	Howard and Howard, 2016 [47]; Tian et al., 2019 [48]
	Groundwater Depletion		Groundwater pumping	Groundwater pumping	Hughes et al., 2020 [1]
Soil Environment Challenges	Soil Erosion	Construction and maintenance of BRI transportation, such as road	Construction and maintenance of BRI energy infrastructure, such as hydropower	Intensive livestock systems	Hughes, 2019 [32]; Benintendi et al., 2020 [49]; Teo et al., 2019 [9]
	Soil Pollution	Rail and airport construction	Pipeline; power line		Ascensão et al., 2018 [36]; Foggin et al., 2021 [44]
Atmospheric Environment Challenge	Global Warming	Global greenhouse gas (GHG) emissions caused by logistics operation; clearing of forests for roads and railway lines; cement production to build roads	Global greenhouse gas (GHG) emissions caused by energy-intensive industries (such as mining, iron, and steel); energy consumption (excessive adoption of fossil fuel and renewable energy); coal-fired power plants; pipeline construction	Intensive livestock systems; microbial decay; burning of plants and organic matters of soil	Rauf et al., 2020 [50]; Ashraf et al., 2022 [51]; Akbar et al., 2020 [52]; Saud et al., 2019 [54];
Geological Environment Challenges	Overexploitation of Resources		Sand and limestone for production of concrete and cement, and fossil fuels for BRI energy projects		Hughes, 2019 [32]; Ascensão et al., 2018 [36]
	Impact on Coastline	Construction of ports; shipping		Reclamation; construction of agricultural, and aquaculture parks	Hughes et al., 2020 [1]; Moores et al., 2019 [60]

## Data Availability

Not applicable.
